# Focusing Bistatic FMCW SAR Signal by Range Migration Algorithm Based on Fresnel Approximation

**DOI:** 10.3390/s151229910

**Published:** 2015-12-21

**Authors:** Yake Li, Siu O’Young

**Affiliations:** Faculty of Engineering and Applied Science, Memorial University of Newfoundland, 230 Elizabeth Avenue, St. John’s, NL A1C5S7, Canada; oyoung@mun.ca

**Keywords:** bistatic FMCW SAR, SAR signal spectrum, range migration algorithm

## Abstract

Frequency modulated continuous wave (FMCW) technique has recently been employed by synthetic aperture radar (SAR) to decrease the radar cost and volume. However, the operation range is limited by the direct energy leakage from the transmitting channel to receiving channel due to the operation principle of FMCW technique. Bistatic configuration is an efficient way to increase the isolation between the transmitter and receiver, which could significantly increase the radar standoff range. A bistatic FMCW SAR spectrum model is proposed by using the Fresnel approximation in this paper. This model is similar to that of a monostatic FMCW SAR spectrum, which allows the existing imaging algorithms to be used on bistatic image processing. Based on the new model and the characteristics of FMCW signal, a modified range migration algorithm (RMA) for FMCW SAR is proposed to focus the image, which requires less memory and computational load than the traditional RMA. Point-target simulation is used to verify the proposed spectral model and real data processing verified the effectiveness of the proposed RMA.

## 1. Introduction

A Frequency modulated continuous wave (FMCW) radar constantly transmits and receives signals and is thus capable of maintaining a high signal to noise ratio with much less peak power than a corresponding pulse radar system. This working principle is readily compatible with the modern solid state devices and hence can greatly decrease the cost and volume of the FMCW radar system. The FMCW technique has recently been used in high resolution synthetic aperture radar (SAR). Several experimental systems have been reported [1,2,3,4,5,6,7,8,9,10].

One limitation of FMCW SAR is the operation distance. Because of the continuous working manner of the FMCW SAR, the energy will leak directly from the transmitter to receiver, which limits the radar standoff range. By separating the transmitting and receiving antennas, a better isolation up to 60 dB [11] could be reached. However, the operation range of FMCW SAR is still limited to several kilometers because the compact size of a monostatic FMCW SAR does not allow much space separation between the transmitting and receiving antennas. Therefore, the transmitted power of a monostatic FMCW SAR is normally limited to several watts even though the devices can handle more.

Bistatic configuration provides a possibility to significantly increase the antennas space isolation while still keeping the small size of the radar. The two-dimensional spectrum of bistatic FMCW SAR has been researched in [12,13]. In [12], an approximated slant range equation is used to express the demodulated signal. The spectrum is then obtained by treating the azimuth frequency as two parts caused by transmitter and receiver separately. Liu *et al.* [13] uses a more accurate slant range approximation which considers the moving of the receiver during the wave propagation. Two separated square roots are obtained in the two-dimensional spectrum due to the consideration of the separately introduced azimuth Doppler frequency by transmitter and receiver. 

The main difficulty of pulse bistatic SAR imaging comes from the dual square roots form of the instantaneous slant range, which changes the range equation from the hyperbola to a flattop hyperbola [14] and thus invalidated most pulse SAR imaging algorithms. The situation is complicated in the FMCW bistatic case by the long duration of the FMCW signal. The in-chirp Doppler problem in monostatic FMCW SAR is proposed in [15], but the situation is more complex in bistatic FMCW SAR due to the dual square roots. 

In this paper, the Fresnel approximation [16] is used to approximate the dual square roots in bistatic FMCW case to a single monostatic-like square root so that the existing imaging ideas in FMCW SAR processing could be applied to bistatic FMCW SAR. 

The other contribution of this paper is a modified range migration algorithm (RMA) for FMCW SAR signal processing. RMA [17,18] is a widely used SAR imaging algorithm well accepted as one of the most accurate SAR imaging algorithms. The original RMA is from seismic processing and then applied [17,18] and extended [19] in pulse SAR image processing. The RMA is introduced into FMCW SAR in [20] by using a more accurate slant range expression. The RMA is one of the preferred algorithms in FMCW SAR processing because the dechirp-on-receive [21] readily brings the raw data to the equivalent range frequency domain, which reduces one range Fourier transform (FT). Moreover, due to the special signal characteristics of the FMCW SAR, the application of RMA could be made more efficient by using a modified RMA introduced in this paper. The modified RMA is proposed based on the novel monostatic-like spectrum obtained by using Fresnel approximation. It reduces the data size needed in the traditional RMA, which improves the processing speed and reduces the memory needed. The modified RMA also generates better images than the traditional RMA if the same size of data is used. The proposed RMA is also effective in monostatic FMCW SAR signal processing.

One drawback of the RMA (both the proposed RMA and the traditional RMA) is that it is sensitive to the motion error of the platform. The motion error introduced to the raw data will also be modified by the Stolt mapping, which causes difficulties for the motion compensation. The related research about motion compensation in RMA can be found in [22,23].

The paper is organized as follows. The FMCW principle is first analyzed in Section 2, which provides one basis for the modified RMA. In Section 3, the monostatic-like spectrum for bistatic FMCW SAR is derived by using Fresnel approximation. Section 4 proposes the modified RMA based on the spectrum obtained in Section 3. Section 5 first uses point-target simulation to verify the proposed spectrum and the modified RMA. Real data are then used to verify the proposed RMA on monostatic FMCW SAR signal processing. Section 6 gives the conclusion.

## 2. FMCW Principle 

**Figure 1:**
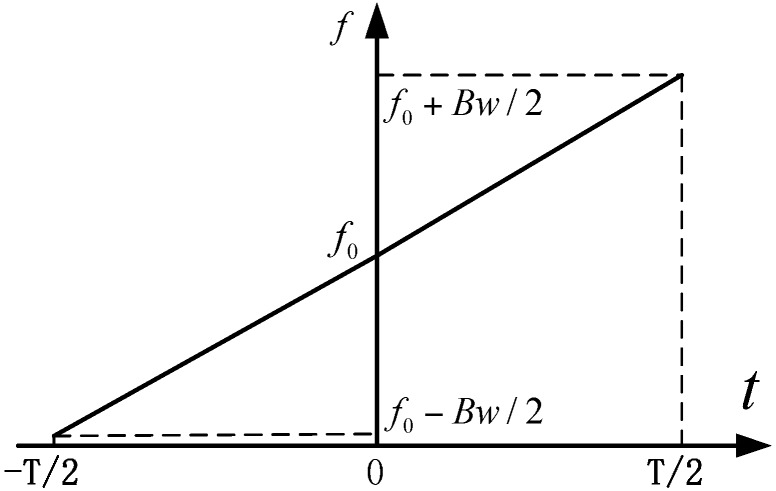
Time frequency plot of a sawtooth sweep chirp.

Figure 1 shows the time frequency plot of a sawtooth modulated FMCW signal, which could be expressed as

(1) $$s_{T}\left(t\right)=rect\left(\frac{t}{T}\right)\exp\left(j\pi kt^{2}+j2\pi f_{0}t\right)$$

where

(2) $$rect(\frac{t}{T})=\{\begin{array}{cc} 1 & \left|t\right|\leq T/2\\ 0 & others \end{array}$$

is the rectangular function. The period of the signal is T, the center frequency is $f_{0}$, the bandwidth is Bw and k=Bw/T is the frequency modulation (FM) rate. The received echo is a time delayed version of Equation (1), which is

(3) $$s_{R}\left(t\right)=rect\left(\frac{t}{T}\right)\exp\left[j\pi k{\left(t-\tau \right)}^{2}+j2\pi f_{0}\left(t-\tau \right)\right]$$

where $\tau =2R_{0}/c$ ($R_{0}$ is the target distance and c is the speed of light) is the two-way time delay of the signal. Considering the speed of light is very large and the distance is normally less than tens of kilometers, the time delay is very small and can be neglected in the rectangular function but only considered in phase [24]. The received signal in Equation (3) is then mixed with the transmitted signal in Equation (1) to generate the intermediate frequency (IF) signal, which is expressed as

(4) $$s_{IF}\left(t\right)=rect\left(\frac{t}{T}\right)\exp\left(j2\pi k\tau t-j\pi k\tau^{2}+j2\pi f_{0}\tau \right)$$

The FT of Equation (4) then gives the distance measurement, which is

(5) $$S_{IF}\left(f\right)=T\text{sinc}\left[T\left(f-k\tau \right)\right]e^{j2\pi f_{0}\tau }e^{-j\pi k\tau^{2}}$$

where sinc(x)=sin(πx)/πx. Equation (5) implies that the FT result of the IF signal is a peak located at f=kτ Hz. If the analog to digital converter (ADC) samples the IF signal during the whole duration of T, then each point in the digital frequency domain represents Δf=1/T Hz. Therefore, the center of the sinc function will be located at

(6) $$\frac{k\tau }{\Delta f}=\frac{\left(Bw/T\right)\cdot \left(2R_{0}/C\right)}{\left(1/T\right)}=\frac{R_{0}}{\left(C/\left(2\cdot Bw\right)\right)}=\frac{R_{0}}{\rho_{s}}\left(\text{points}\right)$$

where $\rho_{s}=C/\left(2\cdot Bw\right)$ is the range resolution of the signal. Equation (6) shows that the change of a distance equaling to the radar resolution will cause one point shift of the sinc function in the discrete frequency domain.

The 3 dB width of the sinc function shown in Equation (5) equals the reciprocal of the coefficient of the variable f [25], which is

(7) $$\frac{1}{T}\left(Hz\right)=1\left(\text{point}\right)$$

Therefore, the resolution of the IF signal after dechirp-on-receive is totally determined by the length of the signal providing that the original signal before demodulation is continuous in this length. 

## 3. Two-Dimensional Spectrum of Bistatic FMCW SAR Based on Fresnel Approximation

**Figure 2:**
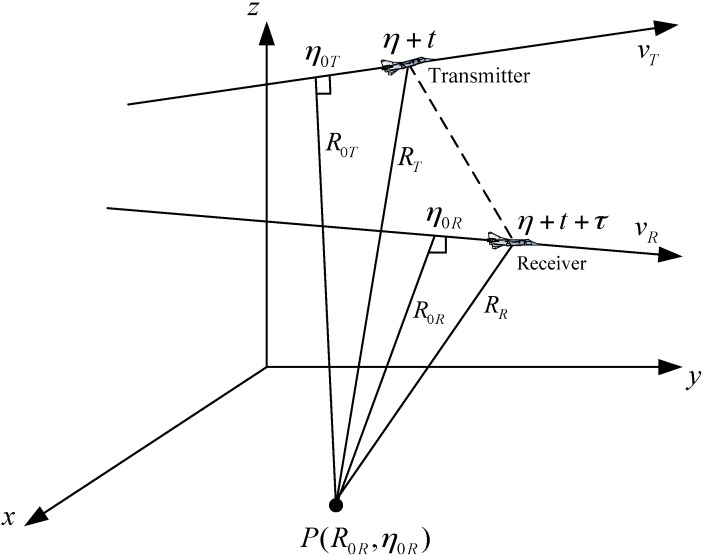
Geometry of bistatic frequency modulated continuous wave (FMCW) synthetic aperture radar (SAR).

The geometry of the general bistatic FMCW SAR is shown in Figure 2. $P\left(R_{0R,}\eta_{0R}\right)$ is a point target in the imaging scene. The transmitter moves with a constant speed $v_{T}$, and the receiver moves with speed $v_{R}$. The closet approach between transmitter and target is $R_{0T}$ (closest approach range) occurring at $\eta =\eta_{0T}$ (closest approach time), and is $R_{0R}$ between the receiver and the target when $\eta =\eta_{0R}$, where η is azimuth time (slow time). Due to the longer pulse duration in FMCW SAR, the traditional stop-and-go assumption is no longer a good approximation, and the movement of the aircrafts inside the pulse needs to be considered [15,26]. Therefore, in Figure 2, the transmitter begins to transmit a certain frequency at time η+t, where t is the fast time. After time τ, the transmitted wave arrives at the receiver. Therefore, the total time used for the propagation is

(8) $$\tau \left(t,\eta \right)=\frac{R_{R}+R_{T}}{c}=\frac{1}{c}\left(\sqrt{R_{0R}^{2}+v_{R}^{2}{\left(\eta +t+\tau -\eta_{0R}\right)}^{2}}+\sqrt{R_{0T}^{2}+v_{T}^{2}{\left(\eta +t-\eta_{0T}\right)}^{2}}\right)$$

A good approximation that keeps the two square roots in Equation (8) symmetric is to neglect τ in the first square root on the right hand side of Equation (8), which means neglecting the movement of the receiver during the propagation of the transmitted wave. The error of this neglecting is normally a few millimeters in airborne applications [27], which is valid for most airborne SAR cases. A more accurate approximation for the slant range is found in [13]. The slant range after approximation is

(9) $$R\left(t,\eta \right)=R_{R}+R_{T}=\sqrt{R_{0R}^{2}+v_{R}^{2}{\left(\eta +t-\eta_{0R}\right)}^{2}}+\sqrt{R_{0T}^{2}+v_{T}^{2}{\left(\eta +t-\eta_{0T}\right)}^{2}}$$

By first squaring R(t,η) of Equation (9) we have

(10) $$\begin{array}{l} R^{2}\left(t,\eta \right)={R_{0R}}^{2}+{R_{0T}}^{2}+{v_{R}}^{2}{\left(\eta +t-\eta_{0R}\right)}^{2}+{v_{T}}^{2}{\left(\eta +t-\eta_{0T}\right)}^{2}\text{+}\\ 2\sqrt{{R_{0R}}^{2}{R_{0T}}^{2}+{R_{0R}}^{2}{v_{T}}^{2}{\left(\eta +t-\eta_{0T}\right)}^{2}+{R_{0T}}^{2}{v_{R}}^{2}{\left(\eta +t-\eta_{0R}\right)}^{2}+{v_{R}}^{2}{\left(\eta +t-\eta_{0R}\right)}^{2}{v_{T}}^{2}{\left(\eta +t-\eta_{0T}\right)}^{2}} \end{array}$$

For long range operation and narrow antenna beamwidth, we have $R_{0R}\gg \left(\eta +t-\eta_{0R}\right)$ and $R_{0T}\gg \left(\eta +t-\eta_{0T}\right)$, thus the last term in the square root of Equation (10) can be neglected. Applying the Fresnel approximation [16] to the square root and then taking the square root of both sides of Equation (10) (R(t,η) is always positive), we obtain Equation (11) after some manipulations.

(11) $$R\left(t,\eta \right)=2\sqrt{R_{0}^{2}+v^{2}{\left(\eta +t-\eta_{c}\right)}^{2}+\delta }$$

where

(12) $$\begin{array}{l} R_{0}=\frac{R_{0R}+R_{0T}}{2}\\ v=\frac{1}{2}\sqrt{\frac{R_{0R}+R_{0T}}{R_{0R}R_{0T}}\beta }\\ \eta_{c}=\frac{R_{0R}\eta_{0T}v_{T}^{2}+R_{0T}\eta_{0R}v_{R}^{2}}{\beta }\\ \delta =\frac{v_{R}^{2}v_{T}^{2}\left(R_{0R}+R_{0T}\right){\left(\eta_{0R}-\eta_{0T}\right)}^{2}}{4\beta }\\ \beta =R_{0R}v_{T}^{2}+R_{0T}v_{R}^{2} \end{array}$$

$R_{0}$, v and $\eta_{c}$ are the equivalent closest approach, velocity and azimuth Doppler center in the new range expression, respectively. Note that v is a function of range in the new model, which is different from the normal airborne SAR cases. Equation (11) is very similar to the monostatic instantaneous slant range expression [27,28] except the last term inside the square root, which is caused by the bistatic configuration. Using the new monostatic-like expression, the two-way propagation delay can now be expressed as

(13) $$\tau \left(t,\eta \right)=\frac{R\left(t,\eta \right)}{c}$$

**Figure 3:**
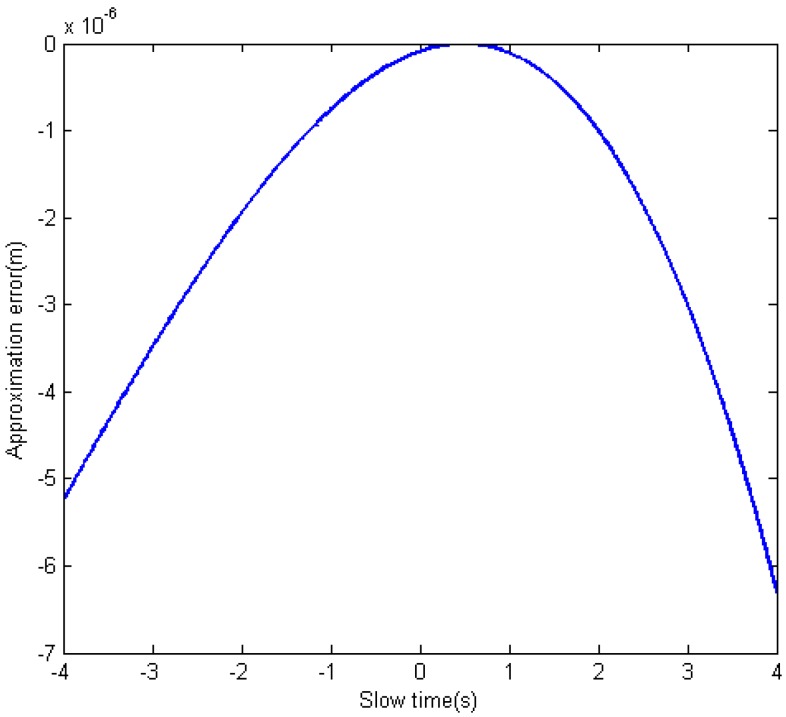
Slant range approximation error.

Figure 3 shows the approximation error when using the Equation (11) to express the original slant range Equation (9). The parameters are shown in Table 1 for a normal FMCW bistatic SAR configuration in which $R_{0R}\gg \left(\eta +t-\eta_{0R}\right)$ and $R_{0T}\gg \left(\eta +t-\eta_{0T}\right)$ are satisfied.

**Table 1:** Parameters for approximation error calculation.

Parameter	Value	Unit
Closest range from receiver to target	16	km
Closest range from transmitter to target	20	km
Receiver speed	50	m/s
Transmitter speed	60	m/s
Closest approach time of receiver	0	s
Closest approach time of transmitter	1	s
Fast time	2	ms

As shown in Figure 3, the maximum approximation error is about $6.3\times 10^{-6}$ m, which will only introduce 0.00126π rad phase error when the center frequency of the transmitted signal is 30 GHz. This phase error is very small and can be neglected. The phase error will be smaller when the center frequency is lower.

The IF signal of the bistatic FMCW SAR after dechirp-on-receive demodulation can be expressed by modifying Equation (4) as

(14) $$s\left(t,\eta \right)=\sigma \left(R_{0R,}\eta_{0R}\right)rect\left(\frac{t}{T}\right)\exp\left(j2\pi f_{0}\tau +j2\pi k\tau t-j\pi k\tau^{2}\right)$$

where $\sigma \left(R_{0R,}\eta_{0R}\right)$ is the reflection coefficient. The last exponential term is known as the residual video phase (RVP), and is normally removed before imaging process. A method that removes RVP is given in [21]. This term is assumed to be removed and will not be included in the following derivation.

Because of the characteristics of the chirp signal, the dechirp-on-receive process has readily brought the signal into the range spectrum domain, hence only one azimuth FT is needed to obtain the two-dimensional spectrum. Perform FT about η in Equation (14), we have

(15) $$S\left(t,f_{\eta }\right)=\sigma \left(R_{0R,}\eta_{0R}\right)rect\left(\frac{t}{T}\right)\int \exp\left(j\phi \left(t,\eta \right)\right)\exp\left(-j2\pi f_{\eta }\eta \right)d\eta$$

where

(16) $$\phi \left(t,\eta \right)=\frac{4\pi }{c}\left(f_{0}+kt\right)\cdot \sqrt{R_{0}^{2}+v^{2}{\left(\eta +t-\eta_{c}\right)}^{2}+\delta }$$

Principle of stationary phase [21,25] can be used at this stage to find the azimuth phase stationary point. By solving $d\left(\phi \left(t,\eta \right)-2\pi f_{\eta }\eta \right)/d\eta =0$, we have

(17) $$\eta =\frac{cf_{\eta }\sqrt{R_{0}^{2}+\delta }}{2v^{2}\left(kt+f_{0}\right)\sqrt{1-\frac{c^{2}f_{\eta }^{2}}{4v^{2}{\left(kt+f_{0}\right)}^{2}}}}+\eta_{c}+t$$

Then the integral in Equation (15) could be approximated and the two-dimensional spectrum can be expressed as

(18) $$S\left(t,f_{\eta }\right)=\sigma \left(R_{0R,}\eta_{0R}\right)rect\left(\frac{t}{T}\right)\exp\left(j\Phi \left(t,f_{\eta }\right)\right)$$

where

(19) $$\Phi \left(t,f_{\eta }\right)=\frac{4\pi \alpha R_{0}}{c}\sqrt{{\left(f_{0}+kt\right)}^{2}-\frac{c^{2}f_{\eta }^{2}}{4v^{2}}}-2\pi f_{\eta }t-2\pi f_{\eta }\eta_{c}$$

and

(20) $$\alpha =\sqrt{1+\frac{v_{R}^{2}v_{T}^{2}}{2R_{0}\beta }{\left(\eta_{0R}-\eta_{0T}\right)}^{2}}$$

The first term in Equation (19) is the equivalent bistatic FMCW SAR square root term. A closer view of the phase shown in Equation (19) could help to understand the major components and the physical interpretation of the equivalent monostatic phase. By expanding the square root of Equation (19) about range time t using Taylor expansion and after some manipulations, we have

(21) $$\Phi \left(t,f_{\eta }\right)=\frac{4\pi \alpha R_{0}}{c}\left[f_{0}D\left(f_{\eta },v\right)+\frac{kt}{D\left(f_{\eta },v\right)}-\frac{k^{2}t^{2}c^{2}f_{\eta }^{2}}{8v^{2}f_{0}^{2}D^{3}\left(f_{\eta },v\right)}+ο\left(k^{2}t^{2}\right)\right]-2\pi f_{\eta }t-2\pi f_{\eta }\eta_{c}$$

where

(22) $$D\left(f_{\eta },v\right)=\sqrt{1-\frac{c^{2}f_{\eta }^{2}}{4v^{2}f_{0}^{2}}}$$

represents the cosine of the instantaneous incidence angle of the receiver. $ο\left(k^{2}t^{2}\right)$ in Equation (21) represents the higher order terms in Taylor expansion. The first term in the square brackets of Equation (21) represents the azimuth modulation. The second term in brackets is the linear function of fast time, which shifts the range sinc function after range FT and represents the azimuth frequency varied range cell migration (RCM). The third term in brackets is the major range-azimuth coupling term, which is normally mentioned as the secondary range compression (SRC) term. The higher order terms are also caused by the range-azimuth coupling and are normally very small in most airborne SAR configurations. However, they could affect the image quality in some extreme cases [27], when they need to be considered and eliminated. It is also the reason that RMA is considered to be a very accurate imaging algorithm because it does not make any approximation to the square root of Equation (19). The second last term is an additional RCM cause by the moving of the radar inside the pulse. The last term is caused by the target’s azimuth position.

## 4. Modified RMA Based on the Bistatic Equivalent Spectrum

The modified RMA follows the traditional RMA steps. The first step is the reference function multiplication (RFM), which focuses the point in the reference range (normally chosen to be the center of the imaging scene). This reference function is

(23) $$\exp\left(j\Phi_{RMF}\left(t,f_{\eta }\right)\right)=\exp\left(-j\frac{4\pi \alpha_{ref}R_{ref}}{c}\sqrt{{\left(f_{0}+kt\right)}^{2}-\frac{c^{2}f_{\eta }^{2}}{4v_{ref}^{2}}}+j2\pi f_{\eta }t\right)$$

**Figure 4:**
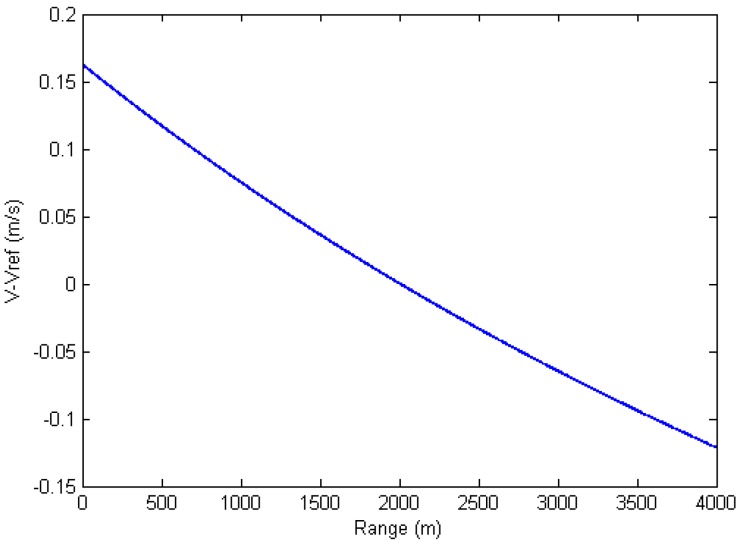
Velocity approximation error.

As shown by Equation (12), the equivalent bistatic velocity varies with range. Therefore, a reference velocity needs to be used in this step. The approximation made in this step is to assume the equivalent bistatic velocity to be the same at all ranges. This is a reasonable approximation because the velocity varies very slowly in long range imaging. Figure 4 shows the speed approximation error using the parameters shown in Table 2. Horizontal axis is the range from the closest edge of the imaging scene.

**Table 2:** Parameters for velocity error calculation.

Parameter	Value	Unit
Closest range from receiver to scene center	16	km
Closest range from transmitter to scene center	20	km
Receiver speed	50	m/s
Transmitter speed	60	m/s

Figure 4 shows that the maximum speed error occurs at the closest edge of the scene, which is a little above 0.15 m/s. The reference velocity used here is 56.1 m/s, thus the maximum speed approximation error is only 0.29% of the reference velocity, which could be neglected.

The second step is the change of variables, which is

(24) $$\sqrt{{\left(f_{0}+kt\right)}^{2}-\frac{c^{2}f_{\eta }^{2}}{4v_{ref}^{2}}}=f_{0}+kt_{1}$$

where $t_{1}$ is the new variable of time. This step is also known as the Stolt mapping, which means a re-mapping of the time axis. If we Taylor expand the square root in Equation (24) and take the first two terms of the series, we have

(25) $$t_{1}=\frac{f_{0}}{k}\left(D\left(f_{\eta },v\right)-1\right)+\frac{t}{D\left(f_{\eta },v\right)}$$

The first term on the right side of Equation (25) is an azimuth frequency varied time shift, which is the major change of the time variable, and the second term is the scaling of time. Since $D\left(f_{\eta },v\right)$ is always less than 1, the change of variable always corresponds to an expansion of the data size in time direction. This expansion could be very large when $D\left(f_{\eta },v\right)$ is significantly smaller than 1, which could dramatically increase the computational load for interpolation and the memory used for calculation. Moreover, the decrease of focus quality occurs as the change in the value of the variable increases [28]. 

The modification of the RMA to decrease the calculation load and improve the image quality takes two steps.

The first step is to modify the mapping formula. Instead of the variable change of Equation (24), the following mapping is used

(26) $$\sqrt{{\left(f_{0}+kt\right)}^{2}-\frac{c^{2}f_{\eta }^{2}}{4v_{ref}^{2}}}=Df_{0}+kt_{1}$$

By making the variable change in Equation (26), the first term on the right side of Equation (25) vanishes, and the mapping only includes the scaling of the time variable. This mapping eliminates the skew of the spectrum caused by parallel time shift. Similar but different variable changes are used in [19,28]. As this Stolt mapping performs all the functions of the traditional one except the azimuth compression, the azimuth modulation needs to be removed after range FT. 

The second step to simplify the RMA is by noticing that the resolution of the IF signal after dechirp-on-receive is only determined by the signal time duration (see Section 2). Therefore, there is no need to perform the whole modified Stolt mapping. The new variable only needs to be limited in the same range of the old time variable, which is

(27) $$-\frac{T}{2}\leq t_{1}\leq \frac{T}{2}$$

By employing the above two modifications in the Stolt mapping, the data size during the mapping can be kept constant, which is important for the low cost FMCW SAR processing.

After the first two steps of the RMA, the signal can be expressed as

(28) $$S_{1}\left(t_{1},f_{\eta }\right)=\sigma \left(R_{0R,}\eta_{0R}\right)rect\left(\frac{t_{1}}{T}\right)\exp\left(j\frac{4\pi \left(\alpha R_{0}-\alpha_{ref}R_{ref}\right)}{c}\left(Df_{0}+kt_{1}\right)-j2\pi f_{\eta }\eta_{c}\right)$$

in which the range and azimuth variables have been successfully separated. The range FT is then performed and the following term is multiplied to finish the azimuth compression

(29) $$\exp\left(j\Phi_{ac}\left(t_{1},f_{\eta }\right)\right)=\exp\left(-j\frac{4\pi \left(\alpha R_{0}-\alpha_{ref}R_{ref}\right)}{c}Df_{0}\right)$$

**Figure 5:**
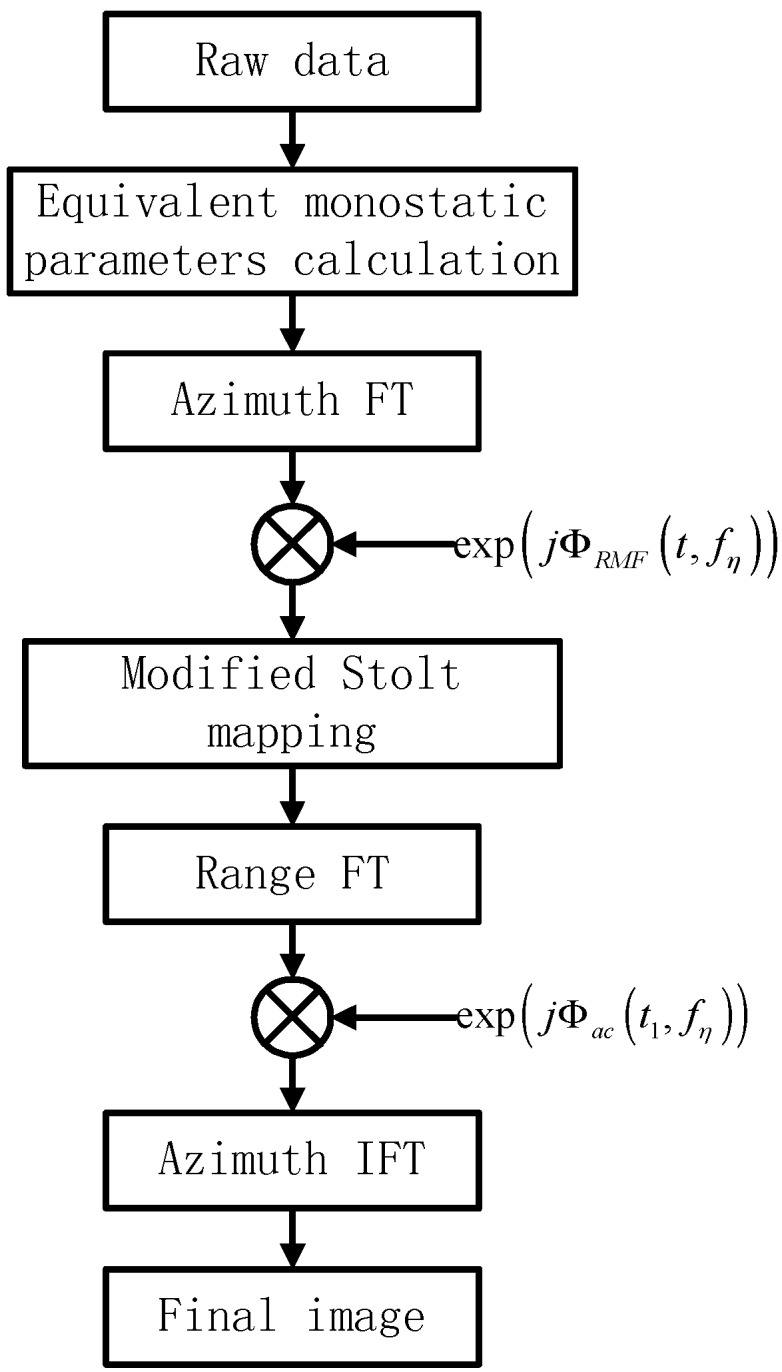
Bistatic FMCW SAR image processing flow diagram.

Then an azimuth IFT finishes the image processing. The whole flow diagram of the bistatic FMCW SAR image processing is shown in Figure 5.

As shown in Figure 5, two phase multiplications and three FT/IFT are needed to finish the processing.

## 5. Results

The proposed FMCW bistatic SAR spectral model and the modified RMA are verified separately in this section.

Section 5.1 uses point-target simulation to verify the proposed two-dimensional spectrum for FMCW bistatic SAR under ideal sensor motion conditions. The modified RMA is also proved by the simulation in this section to be effective in FMCW bistatic SAR signal processing. Extended-scenes simulation is not used in this section. It can be either implemented in time domain (which is very time consuming) [29] or in spectral domain [30,31]. The extended-scenes simulation in spectral domain with non-ideal SAR motion can be found in [32,33].

Section 5.2 uses real data collected by a monostatic FMCW SAR system to prove the effectiveness of the proposed RMA in FMCW SAR image processing. 

### 5.1. Simulation

Point-target simulation is used in this section to verify the proposed equivalent monostatic-like spectrum and the modified RMA. The separation provided by bistatic configuration makes the FMCW SAR possible to operate at long ranges. The simulation parameters are shown in Table 3.

**Table 3:** Simulation parameters.

Parameter	Value	Unit
Closest range from receiver to scene center	20.48	Km
Closest range from transmitter to scene center	23.48	Km
Receiver speed	50	m/s
Transmitter speed	60	m/s
Center frequency	5	GHz
Signal bandwidth	7.5	MHz

**Figure 6:**
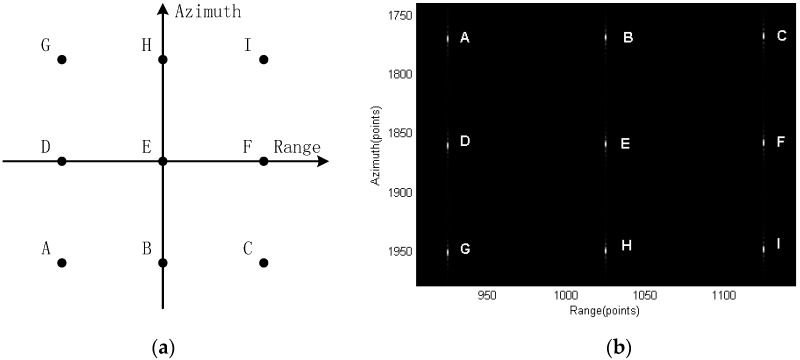
Nine targets in the scene: (**a**) targets position and (**b**) the targets images using the proposed signal model and RMA.

Nine point targets are set in the imaging scene as shown in Figure 6a. Point E is the scene center. Figure 6b shows the processing result using the proposed equivalent spectrum and the modified RMA.

**Figure 7:**
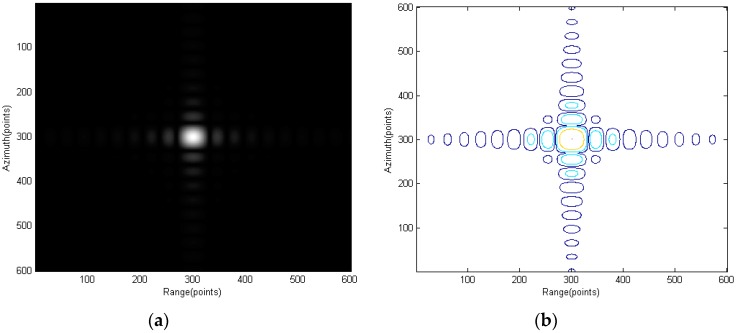
Point E: (**a**) amplitude plot and (**b**) contour plot.

**Figure 8:**
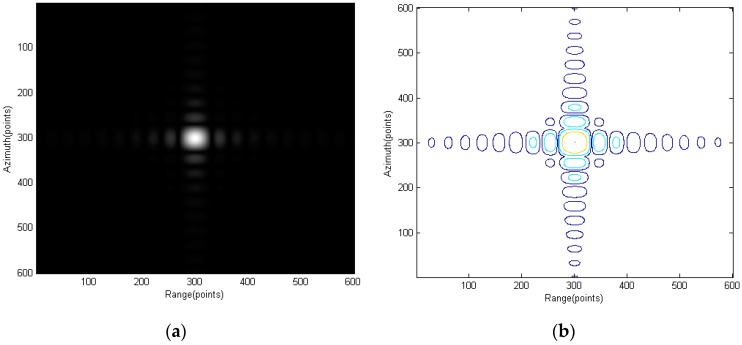
Point G: (**a**) amplitude plot and (**b**) contour plot.

**Figure 9:**
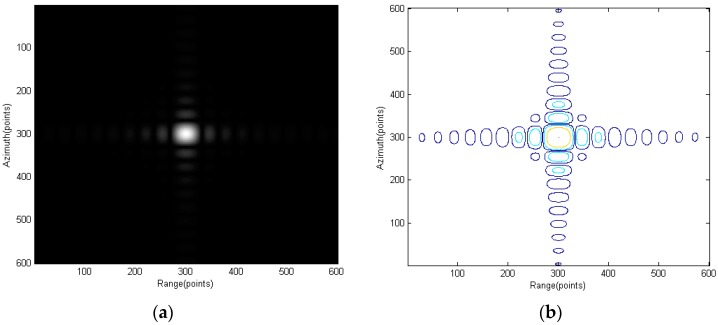
Point C: (**a**) amplitude plot and (**b**) contour plot.

Figure 7 shows the scene center point and Figure 8 and Figure 9 show the points further from center. Interpolation is used to give a better view of the mainlobe and sidelobes of the points. As can be seen from Figure 7 to Figure 9, focus qualities are good and there are very little differences among the three different targets in contour plots while the magnitude plots look the same.

**Figure 10:**
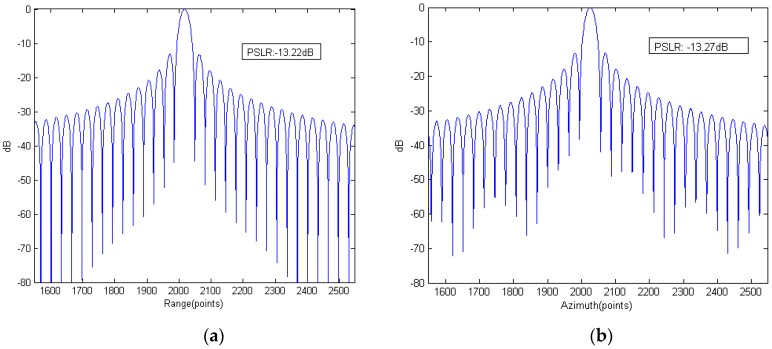
Range and azimuth slices of point C: (**a**) range slice and (**b**) azimuth slice.

Figure 10 shows the range slice and azimuth slice of point C. The PSLR (peak to sidelobe ratio) of range and azimuth slice are a little different, but all fit well with the PSLR of a sinc function. The range 3 dB mainlobe width is 29 samples and the azimuth 3 dB mainlobe width is 30 samples, which mean the range and azimuth resolution are the same.

**Figure 11:**
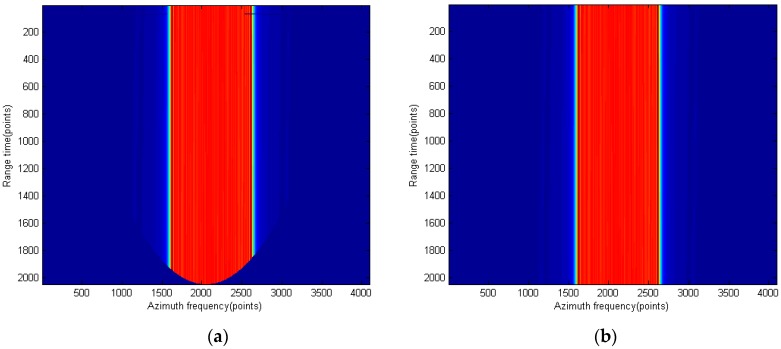
Spectrum comparison after Stolt mapping: (**a**) traditional Stolt mapping and (**b**) proposed Stolt mapping.

Figure 11 shows the comparison of the spectrums after Stolt mapping when using the modified RMA and the traditional RMA. The data sizes used in both spectrums after Stolt mapping are the same. Figure 11a shows that the spectrum is skewed because of the traditional Stolt mapping, which will cause the decrease of the compression quality. A way to solve this problem is to expand the data size and perform the full Stolt mapping, which will of course increase the computational load and the needed memory size. Figure 11b is the result spectrum of the proposed Stolt mapping, which occupies the whole time domain. According to the discussion in Section 2, the range FT of Figure 11b will maintain the same resolution as the spectrum before the Stolt mapping.

**Figure 12:**
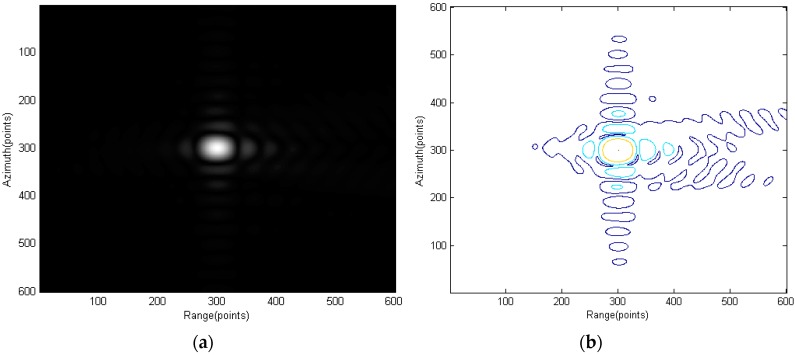
Compression result using the spectrum of Figure 11a: (**a**) amplitude plot and (**b**) contour plot.

Figure 12 shows the compression result using the spectrum of Figure 11a (traditional RMA). A decrease in the image quality can be observed especially from the contour plot.

### 5.2. Real Data Results

Real data collected by a C-band FMCW SAR are used in this section to prove the effectiveness of the proposed RMA in FMCW SAR signal processing. The parameters of the real data collection are shown in Table 4.

**Table 4:** SAR parameters.

Parameter	Value	Unit
Bandwidth	150	MHz
Carrier frequency	5590	MHz
Chirp repetition frequency	250	Hz
SAR speed	60	km/s

**Figure 13:**
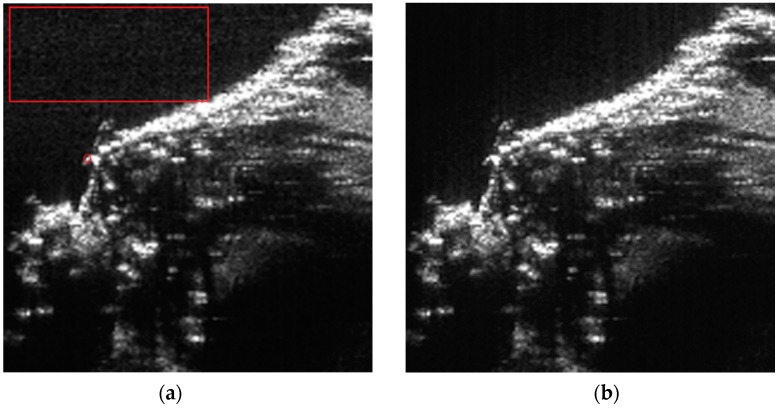
Real data processing results: (**a**) using traditional range migration algorithm (RMA) and (**b**) using proposed RMA.

Figure 13 shows the processed images using the traditional RMA and the proposed RMA. Eight orders of interpolation are used in the Stolt mapping of traditional RMA and in the modified Stolt mapping of the proposed RMA. The spectral sizes (after Stolt mapping) used in both RMAs are the same. No weighting is applied in either the range direction or the azimuth direction for better reveal of the focusing quality. Vertical is the range direction and horizontal is the azimuth direction.

As can be seen in Figure 13, the image processed by the traditional RMA has higher background noise than the image generated by the proposed RMA. The noise is easier to be observed in the red box area (the red box is not drawn in Figure 13b). The red box area of Figure 13b is purer than that of Figure 13a where lots of gray dots are presented. This proves that the image quality obtained using the proposed RMA is better than the traditional RMA.

**Figure 14:**
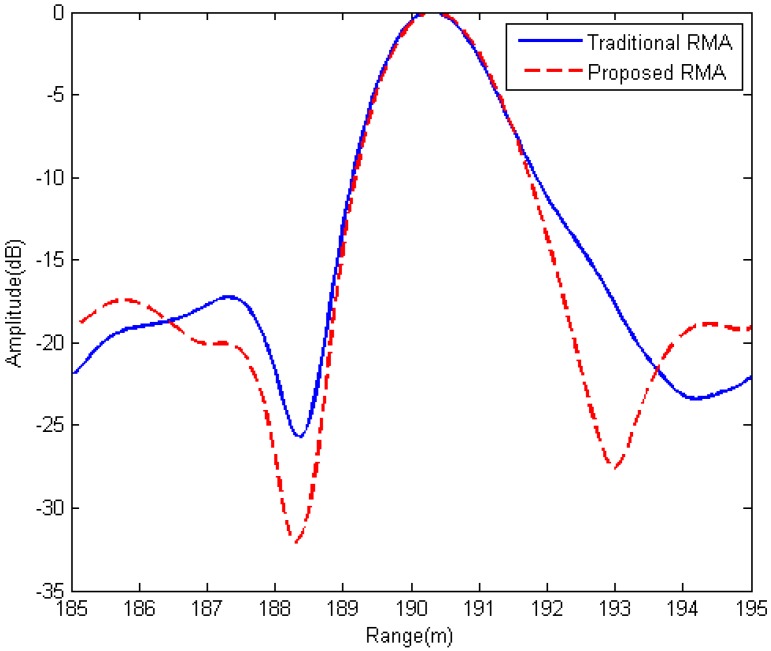
Comparison of the range profiles of an isolated strong point target marked by the red circle in Figure 13a.

The range slices of the isolated strong point target marked by the small red circle in Figure 13a are shown in Figure 14.

In Figure 14, the solid line is the range slice of the marked point in Figure 13a while the dashed line is that of the same point in Figure 13b. An improvement in the range focusing quality (the dashed line has narrower mainlobe) of the proposed RMA than the traditional RMA is observed. This is because the result spectrum of the Stolt mapping in the proposed RMA is not skewed and hence more bandwidth is used to process the image as addressed in Section 3.

## 6. Conclusions

The combination of FMCW SAR and bistatic configuration makes good significance because it provides a reasonable way to solve the intrinsic drawback of FMCW SAR. This paper first proposed a two-dimensional spectrum model for bistatic FMCW SAR based on Fresnel approximation and then proposed a modified RMA for processing the FMCW SAR data. 

The advantage of the proposed spectrum model is that it is very similar to the monostatic FMCW SAR spectrum and thus the existing FMCW SAR imaging algorithms can be used to process bistatic FMCW SAR image. The given spectrum model is accurate under small squint and long range SAR working conditions.

The proposed RMA takes advantage of the unique characteristics of the IF signal in dechirp-on-receive FMCW radar system and decreases the computational load and the memory needed for image processing. Moreover, the proposed RMA has the same accuracy and range of applications as the traditional RMA, which makes it a good alternate to the traditional RMA in FMCW SAR signal processing. When using the same size of spectrum, an improvement of the focusing quality is also obtained. This modified RMA also works in monostatic FMCW SAR processing. Point-target simulations verified the proposed monostatic-like spectrum and real data results proved the effectiveness of the modified RMA.

## References

[1] Edrich M. Design Overview and Flight Test Results of the Miniaturised SAR Sensor MISAR. Proceedings of the First European Radar Conference 2004 EURAD.

[2] Edrich M. (2006). Ultra-lightweight synthetic aperture radar based on a 35 GHz FMCW sensor concept and online raw data transmission. IEE Proc. Radar Sonar Navig..

[3] Meta A., Wit J., Hoogeboom P. Development of a High Resolution Airborne Millimeter Wave FM-CW SAR. Proceedings of the First European Radar Conference 2004 EURAD.

[4] Zaugg E.C., Hudson D.L., Long D.G. The BYU uSAR: A Small, Student-Built SAR for UAV Operation. Proceedings of the IEEE International Conference on Geoscience and Remote Sensing Symposium (IGARSS).

[5] Berizzi F., Martorella M., Cacciamano A., Capria A. (2008). A Contrast-Based Algorithm for Synthetic Range-Profile Motion Compensation. IEEE Trans. Geosci. Remote Sens..

[6] Essen H., Stanko S., Sommer R., Johannes W., Wahlen A., Wilcke J., Hantscher S. Millimetre Wave SAR for UAV Operation. Proceedings of the 2011 Asia-Pacific Microwave Conference.

[7] Max F., Wahlen A., Peter W., Erich M. Processing of MIRANDA35 FMCW-SAR Data using a Time-Domain Algorithm. Proceedings of the 10th European Conference on Synthetic Aperture Radar (EUSAR).

[8] Rossum W.V., Otten M., Dorp P.V. Multichannel FMCW SAR. Proceedings of the 9th European Conference on Synthetic Aperture Radar (EUSAR).

[9] Hermann R., Marc-Michael M., Michael K., Ralph M. Experiences with an Experimental Car controlled by a 77 GHz Radar Sensor. Proceedings of the International Radar Symposium.

[10] Edwards M., Madsen D., Stringham C., Margulis A., Wicks B., Long D. MicroASAR: A Small, Robust LFM-CW SAR for Operation on UAVs and Small Aircraft. Proceedings of the IEEE International Geoscience and Remote Sensing Symposium(IGARSS).

[11] Stove A.G. (1992). Linear FMCW radar techniques. Proc. Inst. Electr. Eng. F—Radar Signal Process..

[12] Liu Y., Deng Y., Wang R., Loffeld O., Wang X. (2013). Model and signal processing of bistatic frequency modulated continuous wave synthetic aperture radar. IET Radar Sonar Navig..

[13] Liu Y., Wang R., Deng Y., Loffeld O. (2013). Bistatic FMCW SAR Signal Model and Imaging Approach. IEEE Trans. Aerosp. Electron. Syst..

[14] Loffeld O., Nies H., Peters V., Knedlik S. (2004). Models and useful relations for bistatic SAR processing. IEEE Trans. Geosci. Remote Sens..

[15] de Wit J.J.M., Meta A., Hoogeboom P. (2006). Modified range-Doppler processing for FM-CW synthetic aperture radar. IEEE Geosci. Remote Sens. Lett..

[16] Geng X., Yan H., Wang Y. (2008). A two-Dimensional Spectrum Model for General Bistatic SAR. IEEE Trans. Geosci. Remote Sens..

[17] Cafforio C., Prati C., Rocca F. (1991). SAR data focusing using seismic migration techniques. IEEE Trans. Aerosp. Electron. Syst..

[18] Rocca F., Prati C., Monti-Guarnieri A. (1989). New Algorithms for Processing of SAR Data.

[19] Reigber A., Alivizatos E., Potsis A., Moreira A. (2006). Extended wavenumber-domain synthetic aperture radar focusing with integrated motion compensation. IET Radar Sonar Navig..

[20] Wang R., Loffeld O., Nies H., Knedlik S., Hagelen M., Essen H. (2010). Focus FMCW SAR Data Using the Wavenumber Domain Algorithm. IEEE Trans. Geosci. Remote Sens..

[21] Carrara W., Goodman R., Majewski R. (1995). Synthetic Aperture Radar Fundamentals. Spotlight Synthetic Aperture Radar: Signal Processing Algorithms.

[22] Zhang L., Sheng J., Xing M., Xiong T., Bao Z. (2012). Wavenumber-Domain Autofocusing for Highly Squinted UAV SAR Imagery. IEEE Sens. J..

[23] Xu G., Xing M., Zhang L., Bao Z. (2013). Robust Autofocusing Approach for Highly Squinted SAR Imagery Using the Extended Wavenumber Algorithm. IEEE Trans. Geosci. Remote Sens..

[24] Skolnik M. (1990). Radar Handbook.

[25] Cumming I.G., Wong F.H. (2005). Digital Processing of Synthetic Aperture Radar.

[26] Meta A., Hoogeboom P., Ligthart L.P. (2007). Signal Processing for FMCW SAR. IEEE Trans. Geosci. Remote Sens..

[27] Zaugg E.C., Long D. (2015). Generalized Frequency Scaling and Backprojection for LFM-CW LFM-CW SAR Processing. IEEE Trans. Geosci. Remote Sens..

[28] Vandewal M., Speck R., Süß H. (2007). Efficient and Precise Processing for Squinted Spotlight SAR through a Modified Stolt Mapping. EURASIP J. Adv. Signal Process..

[29] Mori A., De Vita F. (2004). A time-domain raw signal Simulator for interferometric SAR. IEEE Trans. Geosci. Remote Sens..

[30] Franceschetti G., Migliaccio M., Riccio D., Schirinzi G. (1992). SARAS: A Synthetic Aperture Radar (SAR) Raw Signal Simulator. IEEE Trans. Geosci. Remote Sens..

[31] Deng B., Li X., Wang H., Qin Y., Wang J. (2011). Fast Raw-Signal Simulation of Extended Scenes for Missile-Borne SAR With Constant Acceleration. IEEE Geosci. Remote Sens. Lett..

[32] Franceschetti G., Iodice A., Perna S., Riccio D. (2006). SAR Sensor Trajectory Deviations: Fourier Domain Formulation and Extended Scene Simulation of Raw Signal. IEEE Trans. Geosci. Remote Sens..

[33] Franceschetti G., Iodice A., Perna S., Riccio D. (2006). Efficient simulation of airborne SAR raw data of extended scenes. IEEE Trans. Geosci. Remote Sens..

